# Using three-dimensional printed models for trainee orbital fracture education

**DOI:** 10.1186/s12909-023-04436-5

**Published:** 2023-06-22

**Authors:** Martina Rama, Lauren Schlegel, Douglas Wisner, Robert Pugliese, Sathyadeepak Ramesh, Robert Penne, Alison Watson

**Affiliations:** 1 Sidney Kimmel Medical College, Thomas Jefferson University, Philadelphia, PA USA; 2 Jefferson Health Design Lab, Thomas Jefferson University, Philadelphia, PA USA; 3 Cataract and Primary Eye Care, Wills Eye Hospital, Philadelphia, PA USA; 4 Oculoplastic and Orbital Surgery, Wills Eye Hospital, 840 Walnut Street, Suite 910, Philadelphia, PA 19107 USA

**Keywords:** 3D printing, Ophthalmology, Education, Orbital fracture, Ophthalmology training

## Abstract

**Background:**

Three-dimensional printing is an underutilized technology in ophthalmology training; its use must be explored in complex educational scenarios. This study described a novel approach to trainee education of orbital fracture repair utilizing three-dimensional (3D) printed models as a teaching tool.

**Methods:**

Ophthalmology residents and oculoplastic fellows from multiple training institutions underwent an educational session on orbital fractures, learning through four different models. Participants analyzed orbital fractures through computerized tomography (CT) imaging alone and then utilizing CT imaging with the aid of a 3D printed model. Participants completed a questionnaire assessing their understanding of the fracture pattern and surgical approach. After the training, participants were surveyed on the impact of the educational session. Components of the training were rated by participants on a 5-point Likert scale.

**Results:**

A statistically significant difference (p < .05) was found in participant confidence conceptualizing the anatomic boundaries of the fracture and planning the orbital fracture approach for repair of three out of four models on pre-test post-test analysis. On exit questionnaire, 84.3% of participants thought the models were a useful tool for surgical planning, 94.8% of participants thought the models were a useful tool for conceptualizing the anatomic boundaries of the fracture, 94.8% of participants thought the models were a useful tool for orbital fracture training, and 89.5% of participants thought the exercise was helpful.

**Conclusion:**

This study supports the value of 3D printed models of orbital fractures as an effective tool for ophthalmology trainee education to improve understanding and visualization of complex anatomical space and pathology. Given the limited opportunities trainees may have for hands-on orbital fracture practice, 3D printed models provide an accessible way to enhance training.

**Supplementary Information:**

The online version contains supplementary material available at 10.1186/s12909-023-04436-5.

## Background

The orbital and midfacial boney anatomy are complex. Currently, ophthalmology resident education primarily relies on clinical exposure and cadaver dissections as the central teaching tools for orbital fracture repair. However, it takes repeated exposure to fully conceptualize the orbital anatomy and develop the ability to translate the two-dimensional computerized tomography (CT) imaging into a three-dimensional (3D) orbital construct. This learned skill is central to becoming an orbital surgeon and accelerating this capability is invaluable to resident and fellow training.

3D printing technologies have opened new frontiers, complementing healthcare practices, and being utilized for the purpose of surgical planning and trainee and patient education. Previous studies in other surgical disciplines have shown that 3D models can supplement traditional teaching methods and are superior to two-dimensional (2D) imaging alone in teaching complex anatomy [1, 2]. Surgical training using 3D printed models has become increasingly popular in the recent years due to its low cost and effectiveness in improving students’ visual-spatial skills in the teaching-learning process [3].

Unlike other surgical fields, 3D printing is still an untapped resource in ophthalmology with few published studies about 3D printing applications in ophthalmology education [4]. We hypothesize that by providing patient specific 3D printed models from real patient CT scans, we will enable visual and tactile feedback and sensory aids to improve the learning process for trainees, developing necessary conceptual building blocks in their approach to orbital fractures. Here, we describe the use of three-dimensional (3D) printing to create orbital fracture models and implementation of an educational session for trainees.

## Methods

**Fig. 1:**
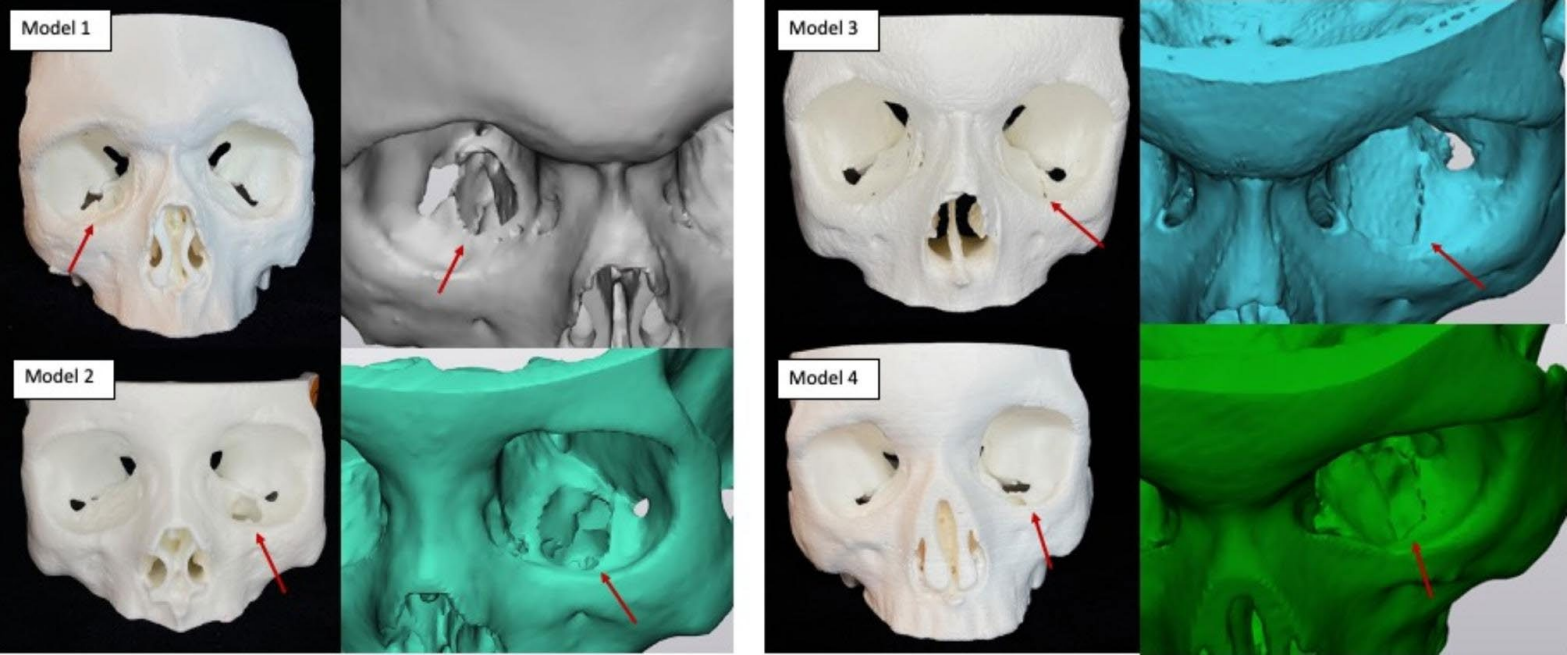
3D printed orbital fracture models and corresponding 3D digital images. The models were printed using white polylactic acid (PLA) plastic filament on an Ultimaker S5 3D printer. Red arrows point to the orbital fracture defect as highlighted in both the printed and zoomed digital model

CT imaging from four patients who presented with orbital fractures was anonymized and exported as a Digital Imaging and Communications in Medicine (DICOM) data set. Three of the fractures were blow-out orbital floor fractures and one was a trap-door fracture. Materialize Mimics Innovation Suite (MIS) software (Materialize, Belgium) was used to create a digital model from the CT scans and make several design customizations including the filling of unnecessary cavities and cropping of the models for printing efficiency and to minimize material cost. The final result was stereolithography (stl) files (Supplemental material 1, see Supplementary Files Legend) that were printed using white polylactic acid (PLA) plastic filament with Polyvinyl acetate (PVA) dissolvable supports on an Ultimaker S5 3D printer (Fig. 1).

Institutional Review Board at Wills Eye Hospital (Philadelphia, Pennsylvania, USA) approved this study and waived consent. All participation was voluntary. This study adhered to the tenets of the Declaration of Helsinki. Prior to hands-on engagement participants completed a survey providing basic demographic information (Supplemental material 2, see Supplementary Files Legend). For each of the four models, participants initially analyzed an anonymized orbital fracture CT scan and completed a questionnaire assessing their understanding of the fracture pattern and surgical approach (Supplemental material 2). Participants were then given a 3D printed model in conjunction with the same previously analyzed CT scan and were asked to fill out the same questionnaire. Our questionnaire also assessed higher level surgical decisions, including what type of implant to use (titanium mesh/titanium-porous polyethylene composite, porous polyethylene sheet, resorbable implant, no implant), whether the implant would need to be fixated, and which ledges were available to maintain the implant into position (posterior, medial, lateral, anterior). Components of the training were rated by participants on a 5-point Likert scale. Participants were randomized to start on one of the four models, and then completed the set moving to the next number model (1-2-3-4-1). An exit questionnaire was administered to assess the overall impact and usefulness of this training exercise (Supplemental material 2, see Supplementary Files Legend). Pre-test post-test analysis was done using paired two sample t-test.

## Results

A total of 20 individuals took part in the study. 30% of participants were PGY2 residents (n = 6), 35% were PGY3 (n = 7), 25% were PGY4 (n = 5), and 10% were oculoplastic fellows (n = 2). None of the participants had participated in an orbital fracture simulation before. Most participants had limited exposure to orbital fracture surgeries, with 45% of participants never having observed an orbital fracture surgery, and 50% having observed one to five orbital fracture surgeries. Only one of the participants had observed 10–15 orbital fracture surgeries. Of note, only 15% of participants had ever performed an orbital fracture surgery as a primary surgeon (n = 3).

There was an increase in participant confidence across many categories between the pre- and posttest surveys (Table 1). Our questionnaire also assessed higher level surgical decisions, including what type of implant to use (titanium mesh/titanium-porous polyethylene composite, porous polyethylene sheet, resorbable implant, no implant), whether the implant would need to be fixated, and which ledges were available to maintain the implant into position (posterior, medial, lateral, anterior). On pretest-posttest analysis of questions regarding implant use and ledge availability none were found to be statistically significant. On exit questionnaire, 84.3% of participants thought the models were a useful tool for surgical planning, 94.8% of participants thought the models were a useful tool for conceptualizing the anatomic boundaries of the fracture, 94.8% of participants thought the models were a useful tool for orbital fracture training, and 89.5% of participants thought the exercise was helpful.

**Table 1:** Pretest-posttest analysis of the questionnaire. Pretest results were collected after the initial analysis of the anonymized orbital fracture CT scan. Posttest results were collected after participants were given a 3D printed model in conjunction with the same CT scan of the orbital fracture. Pretest-posttest analysis was done using paired two sample t-test

	Item	n	Pre-test Mean ± SD	Post-test Mean ± SD	Delta Mean (post-pre)	*P value*
**Model 1**	Q1	18	3.83 ± 0.78	4.28 ± 0.82	0.44	0.028***
	Q2	18	1.88 ± 0.83	2.44 ± 1.33	0.55	0.028***
**Model 2**	Q1	18	3.94 ± 0.91	4.26 ± 1.04	0.32	0.142
	Q2	18	2.26 ± 1.19	2.63 ± 1.07	0.37	0.083
**Model 3**	Q1	20	3.81 ± 0.81	4.57 ± 0.74	0.76	9.88-E05***
	Q2	20	2.86 ± 1.06	3.43 ± 1.36	0.57	0.021***
**Model 4**	Q1	20	3.77 ± 1.19	4.54 ± 0.80	0.77	0.006***
	Q2	20	2.64 ± 1.40	3.04 ± 1.32	0.41	0.029***

Q1: I feel confident conceptualizing the anatomic boundaries of the fracture

Q2: I feel confident planning the orbital fracture reconstruction approach

1 = Strongly Disagree, 2 = Disagree, 3 = Neutral, 4 = Agree, 5 = Strongly Agree

^***^p < .05

## Discussion

Anatomy is inherently a three-dimensional subject, and research has shown that 3D models can enhance trainee understanding of more complex anatomical structures [5–7]. Moreover, it was found that when students have active control over a 3D printed object with the opportunity to observe it from different angles, they are able to better identify anatomic features compared to using the knowledge gained from just viewing the anatomy at multiple orientations [8].

Several studies exist in the literature assessing the use of 3D printed models in facilitating anatomy education in a variety of surgical disciplines. These studies have shown the value of this technology for understanding both soft tissue and bony anatomy alike. Among the soft tissue studies, Lim et al. found that 3D printed hearts were a suitable adjunct to the use of cadaveric materials to teach heart anatomy [9]. Lee et al. showed that 3D printed renal tumor models could help both students and urologists more accurately locate tumors compared to CT alone [10], and Chen et al. found that 3D models of Henle trunk’s variations were an effective tool for students’ understanding of the anatomy, with increased student satisfaction in the learning process. [11] Similarly, boney anatomy studies have highlighted the effectiveness of 3D printed models for teaching purposes, with AlAli et al. finding that 3D models of cleft lip and palate resulted in significant improvement in the percentage of knowledge gained compared to presentation-based educational seminars alone [12], and Wu et al. showing that 3D printed models with spinal, pelvic, and lower limb fractures improved medical students’ understanding of bone spatial anatomy for anatomically complex sites [13].

The anatomy of the orbit, midface, and skull and spatial awareness of the relationships of important structures is complex and challenging for trainees to fully conceptualize. There has been demonstrated value in this regard for the role of 3D printed models in the otolaryngology literature [14]. Most of the 3D printing literature in ophthalmology is around surgical applications, where 3D printed models used as adjuncts for post-traumatic orbital reconstructions were found to reduce soft tissue handling and operation time [15]. All published studies in ophthalmology focus on the use of 3D printed models as an adjunct for orbital fracture reconstruction, rather than a tool for teaching [15]. There was a statistically significant change in confidence after utilizing 3D printed orbital fracture models to conceptualize orbital fracture patterns and plan the surgical approach in 3 out of the 4 models used. For model 2, while a statistically significant pretest-posttest change was not observed, there was still an improvement in confidence. We hypothesize there was limited added value due to fracture’s features; it was the largest of all the fractures and perhaps the most easily conceptualized with imaging alone. This may speak to a potential range of benefits from 3D printed models where some scenarios are more suitable for this learning tool than others.

Pretest and posttest data was also collected regarding the trainees’ ability to make higher level surgical decisions. Specifically, they were asked to determine the type of implant to use and whether the implant may require fixation based on the fracture pattern. We did not observe any significant change in the confidence level for these questions and we hypothesize this is due to lack of exposure at their point in training to an adequate number of surgeries to help inform these more advanced surgical choices (45% of participants observed 0 orbital fracture surgeries). Even with a greater understanding of the fracture and its anatomical boundaries, the trainees may not have known the best approach for implantation and fixation. Therefore, our study confirms that, while valuable, this tool is not a stand-alone resource for trainees but a supplemental tool for enhancement. Even though learner’s ability to make higher level surgical decisions did not change with this training, their confidence in recognizing and anatomically conceptualizing orbital fractures did. We argue that this lays the groundwork for their future learning. We would anticipate that if the study was repeated following further clinical exposure that trainees would be able to utilize the models to help inform higher level surgical decision making. We look forward to future studies, as supported by use in other fields, to employ 3D models for fostering more advanced surgical planning to address these knowledge gaps and provide a tangible mode for practicing implant placement and informing appropriate implant selection.

The primary limitation of this study was the method of model introduction. Participants viewed each one of the available models directly following the CT in random order, therefore for each subsequent case they had the learned experience of matching the CT and the model from the previous pairing. This could result in reduced deltas within the collected data as the trainee may have had enhanced knowledge of fracture pattern conceptualization as they moved through the study. However, we found it important to mimic real-life in which a CT would be the main source of information for developing the treatment plan. Additionally, the utilization of these models for higher surgical decisions should be tested with a larger group of more experienced surgeons. In the future, we plan to expand the use of these models as a teaching tool by assessing their impact on anatomic and spatial relationship understanding as well as surgical planning capabilities. We also look forward to exploring the use of different printing materials to create entrapment models that include bone as well as muscle and soft tissue. These models could aid trainees, who are frequently the first ones evaluating patients, better visualize the relationship between soft-tissue and bony anatomy.

## Conclusion

This study supports the value of 3D printed models of orbital fracture as an effective tool for trainee education, which can serve to improve understanding and visualization of complex anatomical features and fractures. We hope that by sharing these data and the necessary files to allow other institutions to print these models, we will inspire residencies to foster the education of orbital anatomy through the use of 3D models, as it appears to be a currently untapped resource for ophthalmology training programs.

## Electronic supplementary material

Below is the link to the electronic supplementary material.

Supplementary Material 1

Supplementary Material 2

Supplementary Material 3

Supplementary Material 4

Supplementary Material 5

Supplementary Material 6

## Data Availability

The datasets used and/or analyzed during the current study are available from the corresponding author on reasonable request.

## Abbreviations

3D: three-dimensional
CT: computerized tomography
2D: two-dimensional
DICOM: Digital Imaging and Communications in Medicine
MMIS: Materialize Mimics Innovation Suit
PLA: polylactic acid
PVA: polyvinyl acetate
PGY2: post-graduate year 2
PGY3: post-graduate year 3
PGY4: post-graduate year 4

## References

[1] Hu A, Wilson T, Ladak H, Haase P, Fung K (2009). Three-dimensional educational computer model of the larynx: voicing a new direction. Arch Otolaryngol Head Neck Surg.

[2] Knobe M, Carow JB, Ruesseler M (2012). Arthroscopy or ultrasound in undergraduate anatomy education: a randomized cross-over controlled trial. BMC Med Educ.

[3] Graffeo CS, Perry A, Carlstrom LP, et al. 3D Printing for Complex cranial surgery education: technical overview and preliminary Validation Study. *J Neurol Surg Part B Skull Base*. Published online February. 2021;22:–0040. 10.1055/s-0040-1722719. 10.1055/s-0040-1722719 PMC9272238 35832942

[4] Sommer AC, Blumenthal EZ (2019). Implementations of 3D printing in ophthalmology. Graefes Arch Clin Exp Ophthalmol Albrecht Von Graefes Arch Klin Exp Ophthalmol.

[5] Trelease RB (2016). From chalkboard, slides, and paper to e-learning: how computing technologies have transformed anatomical sciences education. Anat Sci Educ.

[6] Chariker JH, Naaz F, Pani JR (2012). Item difficulty in the evaluation of computer-based instruction: an example from neuroanatomy. Anat Sci Educ.

[7] Yuen J (2020). What is the role of 3D Printing in undergraduate anatomy education? A scoping review of current literature and recommendations. Med Sci Educ.

[8] Stull AT, Hegarty M, Mayer RE (2009). Getting a handle on learning anatomy with interactive three-dimensional graphics. J Educ Psychol.

[9] Lim KHA, Loo ZY, Goldie SJ, Adams JW, McMenamin PG (2016). Use of 3D printed models in medical education: a randomized control trial comparing 3D prints versus cadaveric materials for learning external cardiac anatomy. Anat Sci Educ.

[10] Lee JJ, Mills JL. Chronic mesenteric ischemia from diaphragmatic Compression of the Celiac and Superior Mesenteric arteries. Ann Vasc Surg. 2016;30. :311.e5-311.e8. 10.1016/j.avsg.2015.08.001 26541971

[11] Chen Y, Qian C, Shen R (2020). 3D Printing Technology improves medical Interns’ understanding of anatomy of gastrocolic trunk. J Surg Educ.

[12] AlAli AB, Griffin MF, Calonge WM, Butler PE (2018). Evaluating the use of cleft lip and palate 3D-Printed models as a teaching aid. J Surg Educ.

[13] Wu AM, Wang K, Wang JS (2018). The addition of 3D printed models to enhance the teaching and learning of bone spatial anatomy and fractures for undergraduate students: a randomized controlled study. Ann Transl Med.

[14] Leung G, Pickett AT, Bartellas M (2022). Systematic review and meta-analysis of 3D-printing in otolaryngology education. Int J Pediatr Otorhinolaryngol.

[15] Murray-Douglass A, Snoswell C, Winter C, Harris R. Three-dimensional (3D) printing for post-traumatic orbital reconstruction, a systematic review and meta-analysis. *Br J Oral Maxillofac Surg*. Published online July 16, 2022:S0266-4356(22)00201-7. doi:10.1016/j.bjoms.2022.07.001. 10.1016/j.bjoms.2022.07.001 35931592

