# Supplementary material for: Using three-dimensional printed models for trainee orbital fracture education
Source: BMC Med Educ. 2023 Jun 22;23:467. doi: 10.1186/s12909-023-04436-5 (PMC10286337; doi:10.1186/s12909-023-04436-5)
Supplement: Supplementary file 1 — Supplementary Material 1 [file 12909_2023_4436_MOESM1_ESM.docx]

**Questionnaire:**

Demographic Questionnaire:

1. What is your level of training?
   1. MS4
   2. PGY1
   3. PGY2
   4. PGY3
   5. PGY4
   6. Fellow
   7. Attending
2. How many prior orbital fracture wet lab simulations have you attended?
   1. 0
   2. 1
   3. 2
   4. 3
   5. >3
3. How many live orbital fracture surgeries have you observed?
   1. 0
   2. 1-5
   3. 5-10
   4. 10-15
   5. >15
4. How many live orbital fracture surgeries have you performed?
   1. 0
   2. 1-5
   3. 5-10
   4. 10-15
   5. >15
5. If applicable, how many years have you been in practice?

Pre-test and post-test questionnaire:

Answers were rated on a 5-point Likert scale.

1. I feel confident conceptualizing the anatomic boundaries of the fracture.
2. I feel confident planning the orbital fracture reconstruction approach.
3. What type of implant would you use?
   1. Titanium Mesh/Titanium-Porous Polyethylene composite
   2. Porous polyethylene sheet
   3. Resorbable implant
   4. No implant
4. How confident are you in the above answer selection?
5. Would you need to fixate the implant?
   1. Yes
   2. No
6. How confident are you in the above answer selection?
7. If “No” to Q5 “would you need to fixate the implant?” what ledge are available to support and maintain your implant position?
   1. Posterior
   2. Medial
   3. Lateral
   4. Anterior
8. How confident are you in the above answer selection?

Exit Questionnaire:

Answers were rated on a 5-point Likert scale.

1. The 3D printed model are a useful tool for surgical planning.
2. The 3D printed models are a useful tool for conceptualizing the anatomic boundaries of orbital fracture.
3. The 3D printed models are a useful tool for orbital fracture training.
4. Overall, I found this exercise helpful.
