# Supplementary material for: Using three-dimensional printed models for trainee orbital fracture education
Source: BMC Med Educ. 2023 Jun 22;23:467. doi: 10.1186/s12909-023-04436-5 (PMC10286337; doi:10.1186/s12909-023-04436-5)
Supplement: Supplementary file 6 — Supplementary Material 6 [file 12909_2023_4436_MOESM6_ESM.docx]

**Supplementary files legend:**

Name: Sup Material 1 _ model #1

File format: .stl

Title: stereolithography (stl) file Model #1

Description: stereolithography file of Model #1 available for 3D printing

Name: Sup Material 1 _ model #2

File format: .stl

Title: stereolithography (stl) file Model #2

Description: stereolithography file of Model #2 available for 3D printing

Name: Sup Material 1 _ model #3

Format: .stl

Title: stereolithography (stl) file Model #3

Description: stereolithography file of Model #3 available for 3D printing

Name: Sup Material 1 _ model #4

Format: .stl

Title: stereolithography (stl) file Model #4

Description: stereolithography file of Model #4 available for 3D printing

Name: Supplemental material 2

Format: .docx

Title: Questionnaire

Description: Questionnaire administered during the study
